# Supplementary material for: Prevalence of Shigellosis among household contacts of index cases in the EFGH catchment area, Dhaka, Bangladesh
Source: PLoS Negl Trop Dis. 2025 Nov 6;19(11):e0013644. doi: 10.1371/journal.pntd.0013644 (PMC12614799; doi:10.1371/journal.pntd.0013644)
Supplement: Attachment — Submitted filename: Response to Reviewer comments.pdf [file pntd.0013644.s001.pdf]

Point by point response to the reviewer comments

Reviewers' Comments:

Methods

Reviewer #1: The objectives are clear - that is to carry out a family study of shigellosis. However, there is no testable hypothesis. The population could be better described by age, sex, season.

Response: We thank the reviewer for this suggestion. We added a statement to represent the population by age, sex and season. (line 155)

Reviewer #2: The methods are clearly described and are appropriate for addressing the objectives of this study.

Response: We appreciate the reviewer's encouraging comment.

Results

Reviewer #1: The analysis presented match the plan, however, the analysis could have gone into more depth and detail.
i felt the figures 2 and 4 are not needed.

Response: We have removed Figures 2 and 4. Also, we have added a table to represent the data properly, as suggested below. (line 347, Table 1)

Reviewer #2: The results match the analysis plan and are supported by the data presented. The tables and figures are clear and helpfully illustrate the key findings.

Response: Thank you.

Conclusions

Reviewer #1: The authors found many shigella infections in household members of index patients, but it is difficult to know what this means.

Response: We have discussed the significance of the study findings and their implications in the manuscript (lines 224-227, page 11). The study demonstrates a high proportion of asymptomatic Shigella infections among household members of symptomatic patients who seek care. This finding also suggests the possibility of carriers transmitting the disease to the community, particularly in areas with poorly developed water and sanitation systems. The finding will also help policymakers to develop an appropriate preventive strategy to prevent transmission of Shigella in the community.

Reviewer #2: The conclusions are clearly presented and are well-supported by the data provided.

Response: Thank you.

Editorial and Data Presentation Modifications?

Reviewer #1: Editorial comments (the numbers refer to the line numbers).
37, Why mention vaccinations in the abstract when there are no licensed vaccines

Response: We replaced the word ‘vaccination’ with ‘preventive’ in the abstract and discussion (lines 37 and 267; pages 2 and 13).

83. Is there validation that a cold temp is needed. Could it be even harmful to maintain survival of shigellae?

Response: We have followed the SAP of the parent EFGH study, where the temperature was cold while transporting the specimens. We added the reference to the manuscript (page 6, line 101)

85 stool specimens (plural) were…

Response: Corrected.

92 reference(s) needed for the AST assays

Response: Added (page 6, line 110).

98 is this the first time this customized card was used ? is this the same assay as used in the GEMs studies

Response: The TAC cards were customized for the parent EFGH study. We used the same assay used in the GEMS.

114 “All HH stools positive for Shigella were not of the same species or serotype.” This sentence needs to be explained. Not the same as what?

Response: We have modified the sentence for clear understanding (pages 8-9, lines 162-166).

163 Explain “secondary education.” How many years of schooling is this?

Response: Secondary education includes schooling for 5 years, classes from VI to X. We added the definition as a footnote in Table 2.

189 I believe the ERC number should be included.

Response: We have added the ERC number (page 5, line 92).

Reviewer #2: Minor comments/typographical:
Ln 37- I would favor replacing "vaccination" with " prevention" as the paper does not deal at all with vaccination and other prevention strategies are alluded to in the results (i.e reheating meals)

Response: We have replaced the word as suggested.

Ln 136- fill in the % value

Response: We added the % value.

Ln 186- Ethical clearance should go under methods

Response: Replaced in the method section.

Fig 1- "CONSORT" should be capitalized

Response: Done.

Summary and General Comments

Reviewer #1: This is an ambitious study to study shigella infections in households of index patients with shigellosis. It is an interesting study, but it seems the analysis could have gone into more depth in their analysis. For example:

1. When the study on household infections was being considered, did the authors have a hypothesis about the household infections? For example, did they want to determine if the infections were being spread between family members and if so, how? To answer this question, they would need to track the specific strains between household members. Perhaps alternatively, they just wanted to see if index cases identified households that have a high risk for shigellosis in general because of poor water/sanitation. If there was a hypothesis, what was the conclusion after doing the study?

Response: We agree with the reviewer that our study did not begin with a specific hypothesis. However, our aim was to demonstrate the prevalence of asymptomatic infection among the household members of *Shigella* index cases, indicating that index cases identified households have a high risk of shigellosis, which is mentioned in the manuscript now (page 11, lines 223 and 224). Moreover, we identified significant findings that have important implications for public health policy (page 13, lines 265-269).

2. What were the characteristics of the index cases? Did they all have dysentery or did some have watery diarrhea and were classified as an index case only after a positive culture was detected. Did the clinical symptoms change the rate of infections in household members?

Response: All index cases had acute watery diarrhea; around 30% had dysentery. However, we did not find any relationship between the rate of infection among HH contacts and the clinical presentation of the index cases.

3. What were the species and serotypes of the index cases, and how did these match the species / serotype of the infections in the household members. It would seem, there were many different strains of shigella in the household members, but they may not be related to the infection in the index cases.

Response: We have added the number of households with matched serogroups between household members and index cases in the result section (page 9, lines 162-164). We have also incorporated a statement suggested by the reviewer in the discussion (page 11, lines 223-226).

4. What is the rate of shigella infection in the community? In this slum area of the city, it may be that shigella infections are common in the general population, especially infections that can be detected by PCR. Without a comparison of rates in a control group, it is difficult to know if the households had a higher rate than would be expected.

Response: There are no recent community-based data, except for our parent study (EFGH), in which we found a 14.2% isolation rate among children aged 6–35 months. We agree that it is

difficult to determine whether the households had a higher rate, as there was no control group (households without index cases).

5. The analysis of households should be by household as well as individual. How many of the households had an infection. What was the distribution of the numbers of infections in the infected households. Did some households have many people infected and others had 0 or 1? It was not clear if the risk factors looked at households or only at individuals with infections.

Response: 29 households out of 118 had an infection. 2 members from 5 households and 3 members form one HH were infected. We added the statement on page 9, lines 165-166. We have presented the risk factors at the household-level as well as the individual-level (Table 2).

6. I believe Table 1 can better describe the characteristics of the household members by age and sex.

Response: We have described the characteristics of HH members by age and sex/gender for the individual-level analysis.

7. Was there any seasonality among the index cases.

Response: We did not observe any specific seasonality among the index cases in the parent EFGH study. The manuscript has been submitted.

8. I believe the discussion section could be improved by first describing what the authors found in their study. I prefer to understand all the findings from this study first. Then, in later paragraphs compare their findings to other studies.

Response: We have elaborated the first paragraph of the discussion and revised the second paragraph to include comparisons with other studies, based on the reviewer's comment (pages 11, lines 223-226, page 12, lines 227-234).

9. In terms of limitations, I would highlight the lack of control group. Ideally this could have been a case control study in which households of index patients with a non-diarrheal illness could be compared. Obviously, it is too late to do this now, but is there another way to estimate the rates in the community?

Response: We appreciate the reviewer's insightful comment that the study could have been done using the case-control design. Since we have utilized the EFGH platform to conduct this sub-study, we could not plan to include households of index patients with a non-diarrheal

illness as controls. The parent EFGH study was conducted between 2022 and 2024 to determine the burden of *Shigella* among children aged 6-35 months, and the manuscript has been submitted. We acknowledge that there is no other way to estimate the isolation rates in the community.

10. The antibiotic sensitivity patterns must have differed by species and serotype. By lumping together, I believe we do not have an appreciation of the true antibiotic sensitivity patterns.

Response: We have added a new figure and statement of the antibiotic sensitivity patterns for different serogroups (Figure 2).

Figure 2. I do not think a figure is needed. A table would be better.

Response: We removed Figures 2 and 4, added a table to show the findings of culture and TAC testing.

Figure 3. Include the number of isolates tested

Response: We added the number of isolates tested in the figures.

Figure 4. The figure does not have a label for the y axis. With so few pathogens identified with the Taq card, it would seem this does not deserve a figure. The data could be included in the text.

Response: Please see the above response.

Reviewer #2: This manuscript describes a study assessing factors associated with Shigella carriage among household contacts of index children aged 6-35 months presenting with Shigella infection in Dhaka, Bangladesh. The cohort was relatively large, but still of limited size to detect differences in some factors. The methods were rigorously defined and support the conclusions drawn, which showed frequent asymptomatic carriage in household contacts and risk factors that included failure to reheat meals. I admit to being a little confused about how toilet type is shown to be significant in even the univariate analysis despite having only 1 infection in a total 2 individuals without flush toilets.
I think this paper does add to the general knowledge of household transmission of shigella and could be useful in developing strategies for screening or prevention.

Response: We thank the reviewer.
